# Supplementary material for: Feeding and swallowing outcomes of children receiving long-term ventilation: A scoping review protocol
Source: PLoS One. 2024 Feb 22;19(2):e0287872. doi: 10.1371/journal.pone.0287872 (PMC10883525; doi:10.1371/journal.pone.0287872)
Supplement: S2 Checklist — (DOC) [file pone.0287872.s002.doc]

**Completion of PRISMA-P Checklist:**

*From: Shamseer L, Moher D, Clarke M, Ghersi D, Liberati A, Petticrew M, Shekelle P, Stewart L, PRISMA-P Group. Preferred reporting items for systematic review and meta-analysis protocols (PRISMA-P) 2015: elaboration and explanation. BMJ. 2015 Jan 2;349:g7647*

**Title: Feeding and swallowing outcomes of children receiving long-term ventilation: A scoping review protocol**

**Type of Review: Scoping Review**

| **Section** | **Item Number** | **Completed?** |
| --- | --- | --- |
| Administrative Information | 1a | Yes, protocol of *scoping review* |
|  | 1b | N/A |
|  | 2 | Yes |
|  | 3a | Yes |
|  | 3b | Yes |
|  | 4 | N/a |
|  | 5a | Yes |
|  | 5b | Yes |
|  | 5c | Yes |
| Introduction | 6 | Yes |
|  | 7 | Yes, (Participants, Concept, Context (PCC) used as per scoping review protocol) |
| Methods | 8 | Yes, (Participants, Concept, Context (PCC) used as per scoping review protocol) |
|  | 9 | Yes |
|  | 10 | Yes |
|  | 11a | Yes |
|  | 11b | Yes |
|  | 11c | Yes |
|  | 12 | Yes |
|  | 13 | Yes |
|  | 14 | Not required in scoping review |
|  | 15a | Not required in scoping review |
|  | 15b | Not required in scoping review |
|  | 15c | Not required in scoping review |
|  | 15d | Yes (data extraction form presented in Appendix II) |
|  | 16 | Not required in scoping review |
|  | 17 | Not required in scoping review |
