# Supplementary material for: Feeding and swallowing outcomes of children receiving long-term ventilation: A scoping review protocol
Source: PLoS One. 2024 Feb 22;19(2):e0287872. doi: 10.1371/journal.pone.0287872 (PMC10883525; doi:10.1371/journal.pone.0287872)
Supplement: S1 Appendix — Date of search: 13th December 2021. (DOCX) [file pone.0287872.s003.docx]

**Appendix I: Full search strategy for PubMed**

**Date of search: 13^th^ December 2021**

|  | **Search Term** | **Results** |
| --- | --- | --- |
| 1 | “oral feed*” OR “oral-feed” OR “orally feed*” OR “orally-feed*” OR swallow* OR dysphag* OR deglutition OR “feeding behaviour” OR “feeding behavior” OR bottle OR breast OR suck* OR eat OR eating OR drink* OR aspirat* OR oromotor OR “oral motor” OR gastrostomy | 1,209,939 |
| 2 | ventilat* OR “artificial respiration” OR “assisted ventilation” OR “continuous positive airway pressure” OR CPAP OR “bilevel positive airway pressure” OR BiPAP OR “nasal CPAP” OR “nCPAP” OR “nasal continuous positive airway pressure” OR tracheostomy OR tracheotomy | 252,578 |
| 3 | child* OR paediatr* OR pediatr* OR infant OR baby OR babies OR preschool OR newborn OR neonat* OR premature OR preterm | 4,086,547 |
| 4 | #1 AND #2 AND #3 | 3,978 |
| 5 | #4 NOT (“pregnan*” OR analgesic* OR “foreign bod*” OR “tidal volum*” OR “stem cell*” OR azithromycin* OR drug* OR “adrenergic beta-agonists” OR intraoperative OR perioperative OR contamination OR lactoferrin OR segmentography OR “lung ultrasound” OR “in vitro” OR bacteremia OR metabolism OR nitrate* OR “lesion*” OR “synechiae” OR anorexia* OR bulimia OR “binge eating” OR “otitis media” OR eustachian OR puncture OR dental*) | 2000 |
| 6 | #4 (with results filtered by year: 2000-2021) | 1370 |
