# Supplementary material for: Feeding and swallowing outcomes of children receiving long-term ventilation: A scoping review protocol
Source: PLoS One. 2024 Feb 22;19(2):e0287872. doi: 10.1371/journal.pone.0287872 (PMC10883525; doi:10.1371/journal.pone.0287872)
Supplement: S2 Appendix — (DOCX) [file pone.0287872.s004.docx]

**Appendix II: Data Extraction Form**

| **Data Extraction Form** | |
| --- | --- |
| Study Parameters | Citation details (Author/s, year, title, journal, volume, issue, pages) |
|  | Country of origin |
|  | Number of participants |
|  | Age of participants |
|  | Study design |
| Ventilation Parameters | Medical population |
|  | Sub-group of children requiring LTV support |
|  | Non-invasive or invasive ventilation |
|  | Other information about ventilation (i.e. CPAP/BiPAP, duration of use) |
| Feeding and Swallowing Parameters | Type of feeding method (e.g. non-oral / oral / combination) |
|  | Description of feeding and swallowing difficulties |
| Assessment and Intervention Parameters | Assessment type (clinical / instrumental) |
|  | Assessment findings |
|  | Intervention implemented |
|  | Intervention outcomes |
| Health Status Parameters | Impact of feeding and swallowing needs on health status |
| Quality of Life Parameters | Impact of feeding and swallowing needs on quality of life of child and/or family |
